# A randomized, double-blind, placebo-controlled trial to evaluate the hypoglycemic efficacy of the mcIRBP-19-containing *Momordica charantia* L. fruit extracts in the type 2 diabetic subjects

**DOI:** 10.29219/fnr.v66.3685

**Published:** 2022-01-03

**Authors:** Yi-Sun Yang, Nian-Yi Wu, Edy Kornelius, Chien-Ning Huang, Nae-Cherng Yang

**Affiliations:** 1 Department of Internal Medicine, Division of Endocrinology and Metabolism, Chung Shan Medical University Hospital, Taichung, Taiwan; 2 Department of Internal Medicine, School of Medicine, Chung Shan Medical University, Taichung, Taiwan; 3 Department of Nutrition, Chung Shan Medical University, Taichung, Taiwan; 4 Department of Nutrition, Chung Shan Medical University Hospital, Taichung, Taiwan

**Keywords:** bitter gourd extracts, Momordica charantia insulin receptor binding peptide-19, type 2 diabetic subjects, fasting blood glucose, HbA1c

## Abstract

**Background:**

The fruits of *Momordica charantia* L., also named as bitter gourd or bitter melon in popular, is a common tropical vegetable that is traditionally used to reduce blood glucose. A peptide derived from bitter gourd, *Momordica charantia* insulin receptor binding peptid-19 (mcIRBP-19), had been demonstrated to possess an insulin-like effect *in vitro* and in the animal studies. However, the benefit of the mcIRBP-19-containing bitter gourd extracts (mcIRBP-19-BGE) for lowering blood glucose levels in humans is unknown.

**Objective:**

This aim of this study was to evaluate the hypoglycemic efficacy of mcIRBP-19-BGE in subjects with type 2 diabetes who had taken antidiabetic medications but failed to achieve the treatment goal. Whether glucose lowering efficacy of mcIRBP-19-BGE could be demonstrated when the antidiabetic medications were ineffective was also studied.

**Design:**

Subjects were randomly assigned to two groups: mcIRBP-19-BGE treatment group (*N* = 20) and placebo group (*N* = 20), and were orally administered 600 mg/day investigational product or placebo for 3 months. Subjects whose hemoglobin A1c (HbA1c) continued declining before the trial initiation with the antidiabetic drugs were excluded from the subset analysis to further investigate the efficacy for those who failed to respond to the antidiabetic medications.

**Results:**

The oral administration of mcIRBP-19-BGE decreased with a borderline significance at fasting blood glucose (FBG; *P* = 0.057) and HbA1c (*P* = 0.060). The subgroup analysis (N = 29) showed that mcIRBP-19-BGE had a significant effect on reducing FBG (from 172.5 ± 32.6 mg/dL to 159.4 ± 18.3 mg/dL, *P* = 0.041) and HbA1c (from 8.0 ± 0.7% to 7.5 ± 0.8%, *P* = 0.010).

**Conclusion:**

All of these results demonstrate that mcIRBP-19-BGE possesses a hypoglycemic effect, and can have a significant reduction in FBG and HbA1c when the antidiabetic drugs are ineffective.

## Popular scientific summary

- Hypoglycemic efficacy of mcIRBP-19-BGE was evaluated in the type 2 diabetic patients.
- mcIRBP-19-BGE could significantly reduce FBG and HbA1c in the subjects who failed to respond to the antidiabetic drugs.
- The results demonstrated that mcIRBP-19-BGE had a hypoglycemic effect and could be an alternative treatment option for the patients when the antidiabetic drugs were ineffective.

Today, diabetes mellitus (DM) has become a critical issue with about 425 million patients globally in 2017 within the age group of 20–79 years, according to the International Diabetes Federation (1). Currently, the oral hypoglycemic medication is one of the main treatment options for type 2 diabetes. However, there are still about two-thirds of the patients who have failed to achieve the treatment goal, that is, hemoglobin A1c (HbA1c) < 7.0% (2). According to the treatment guidelines, it is suggested that patients should take insulin injection with or without combining oral hypoglycemic medications when they have failed to well control blood glucose levels after taking three or more hypoglycemic medications with different mechanisms (3). Although the treatment combination of oral metformin and insulin injection for 24 weeks is reported to decrease 2.5% of HbA1c (4), many type 2 diabetic patients still refused to get initiated into the treatment due to the fear of injection pain, hypoglycemia, weight gain, and the negative impression of insulin injection (5, 6). Therefore, a natural ingredient with a similar effect as insulin, which can be taken orally, will benefit patients who fail to achieve the treatment goal with oral medications and refuse to inject insulin.

The fruits of *Momordica charantia* L., popularly known as bitter gourd or bitter melon, is a common tropical vegetable that is traditionally being used to reduce blood glucose (7–9). There are at least 228 ingredients that have been verified in *Momordica charantia,* and some of the phytochemicals and proteins among the ingredients may have effects in lowering blood glucose levels (9, 10). For example, charantin found in *Momordica charantia* was demonstrated to be beneficial against diabetes in animal trials (10), and there are four additional triterpenoid compounds that have been demonstrated to activate AMP-activated protein kinase that may be related to the blood glucose lowering mechanisms in *Momordica charantia* (11, 12). More interestingly, proteins in *Momordica charantia*, such as polypeptide-P, M.Cy protein, and MC6 protein, were reported to have the effect of lowering blood glucose levels in animal studies (13–15). In 2013, researchers found that a peptide extracted from *Momordica charantia*, mcIRBP (*Momordica charantia* insulin receptor binding peptide), had 68 amino acids, with a molecular weight of 7 KDa, and could bind with the insulin receptor (16). After further hydrolysis with digestion enzyme, peptides with 19 and nine amino acid sequence peptides were discovered, and were called mcIRBP-19 (*Momordica charantia* insulin receptor binding peptid-19) and mcIRBP-9 (*Momordica charantia* insulin receptor binding peptid-9), respectively. Both peptides were able to bind with insulin receptor, activate the kinase activity and the downstream molecular communicators, and therefore, had the benefit of lowering blood glucose levels (17–19). The results revealed that the peptides possessed the insulin-like effect.

Many animal trials have demonstrated that the extract of *Momordica charantia* and its ingredients are beneficial in lowering blood glucose (10); however, results of the *Momordica charantia* extract in human trials are not consistent (7, 10, 20). For example, two clinical trials reported results showing no effect on controlling blood glucose. One of the trials recruited patients with type 2 diabetes and administered bitter gourd extract capsules for 3 months (21), and the other treated the type 2 diabetic patients with pills made from an entire dried bitter gourd for 1 month (22). In contrast, a randomized, double-blind trial reported that the level of fructosamine in the blood was effectively reduced among newly diagnosed type 2 diabetes patients who were administered *Momordica charantia* fruit extract capsules (1,000 mg/day) for 1 month (8). Another study showed that oral bitter gourd extracts (1,000 mg/day) had a significant reduction in HbA1c in the type 2 diabetic patients (23). Although the testing results for bitter gourd extracts in human studies are not consistent, the investigational product in this study has been characterized as containing mcIRBP-19, which may grant the products more opportunity to have a hypoglycemic effect for type 2 diabetes.

In this study, we intended to explore the benefit of the mcIRBP-19-containing bitter gourd extracts (mcIRBP-19-BGE) for lowering blood glucose in diabetes. However, we could not recruit the type 2 diabetic patients without taking any hypoglycemic medication for ethical considerations. Thus, this human trial was designed to investigate the efficacy and safety of mcIRBP-19-containing bitter gourd extracts (mcIRBP-19-BGE) in type 2 diabetic patients who had failed to achieve the treatment goal under hypoglycemic medication treatment. We hypothesized that the investigational product possesses a hypoglycemic effect when the antidiabetic medications cannot achieve the treatment goal. Because this study adopted an add-on treatment design, the hypoglycemic efficacy of mcIRBP-19-BGE was further assessed in the subset subjects who showed no medication efficacy. The hypoglycemic efficacy was evaluated by fasting blood glucose (FBG) and HbA1c. In addition, several indicators of health conditions for the subjects were also evaluated.

## Methods

### Subjects

This randomized, double-blind, placebo-controlled, parallel comparison study was conducted in the Division of Endocrinology and Metabolism of Chung Shan Medical University Hospital (CSMUH) from May through November 2017. The protocol and study material were approved by the CSMUH Institutional Review Board (IRB), and were registered with the National Institutes of Health, ClinicalTrials.gov identifier: NCT03151837. The major enrollment criterion was that type 2 diabetes patients who had been treated with more than one oral medication but did not achieve the treatment targets (refers to steady dosage treatment for 3 months but still with FBG levels between 140 and 270 mg/dL and HbA1c 7–10%). Patients who had serum creatinine >1.8 mg/dL, serum alanine aminotransferase (ALT), aspartate aminotransferase (AST), total bilirubin, or alkaline phosphatase >2.5 times of normal range, anemia (hemoglobin [Hb] male < 11 g/dL; female < 10 g/dL), severe angina, moderate–severe heart failure with left ventricular hypertrophy, body mass index (BMI) lower than 18 or greater than 38, sudden and recent changes in dietary habits (within 1 month) or weight change exceeding 10%, unstable medical condition or life expectancy of less than 6 months, a known history of allergy to ingredients in the test product, severe diabetes complications or acute disease deemed unsuitable for participation judged by the investigators, being pregnant or breastfeeding, or had been administered any experimental drugs within 30 days were excluded from this study. A total of 41 eligible subjects with written informed consent were randomly assigned to one of the two groups: mcIRBP-19-BGE capsules (600 mg/day) (*n* = 21) and placebo group (starch: 600 mg/day) (*n* = 20). All study subjects underwent screening evaluation up to 7 days prior to administration of the investigational products. The privacy rights of the subjects have been well protected. Patients were also advised to take prescribed hypoglycemic medication under physicians’ instruction since the investigational products were not supposed to replace the regular treatment. In addition, all study subjects were asked to pay attention for better control of their blood glucose through the education in this study, and to maintain a stable diet and lifestyle.

### Subset subjects

Although the enrollment criteria had prevented patients with good response to the hypoglycemic medications, some of the recruited patients still showed a significant response to the antidiabetic drugs. Because the diabetic patients are required to regularly return to the hospital every 3 months for follow up and medications, we could find the patients’ HbA1c at three and 6 months before the enrollment from the medical records. A subset of the study subjects that excluded those whose HbA1c level continuously declined before the enrollment (i.e. the patient’s HbA1c levels at month 3 before treatment were higher than that at the enrollment) were then used to assess the hypoglycemic effect of mcIRBP-19-BGE. The subset analysis also could be used to evaluate the hypoglycemic efficacy of mcIRBP-19-BGE under the ineffective treatment condition from the antidiabetic medications.

### Investigational products

The investigational product, mcIRBP-19-BGE (with the brand name of Insumate^®^; batch number IN161116F01P) was obtained from Greenyn Biotechnology Co., Ltd (Taichung, Taiwan). The investigational product was 100% made from fruits of *Momordica charantia* L. After washing and slicing, the fruits of *Momordica charantia* L. were extracted with 100% water. After filtration, centrifugation, concentration, freeze vacuum drying, pulverization, and sieving of the extracts, mcIRBP-19-BGE was prepared. The industrial product of mcIRBP-19-BGE contains approximately 0.17% of mcIRBP-19. The product has also been tested to contain undetectable levels of heavy metals, plasticizers, or pesticides. Both of the products used in this trial, mcIRBP-19-BGE (300 mg/capsule) and placebo starch (300 mg/capsule), were identical in appearance and were manufactured by ISO certified United Biocaps Corp. (Taichung, Taiwan). The study subjects were suggested to take one capsule before lunch and dinner each day, and not to take within 30 min of other medications. The dosage (i.e. 600 mg/day of mcIRBP-19-BGE) was recommended by the manufacturing company, and it contained approximately 471 nmol of mcIRBP-19 (calculated from the 0.17% mcIRBP-19 in mcIRBP-19-BGE, and the molecular weight of mcIRBP-19 is approximately 2,162 g/mol). According to the previous study (18), single-intraperitoneal administration with approximately 2.5 nmol/kg mcIRBP-19 could significantly enhance the clearance of glucose in diabetic mice. For example, for calculation, the dosage of 471 nmol/day used for a man of 66.8 kg was about 2.82 times higher compared with that used in the animal study (i.e. 167 nmol/66.8 kg per day).

### Outcome assessment

After a 1- to 7-day screening period, the study products were administered orally for 12 weeks. All laboratory samples were collected after an overnight fasting from all subjects. Laboratory tests, including FBG, HbA1c, and safety indicators (i.e. AST, ALT, creatinine, blood urea nitrogen [BUN], uric acid, and Hb), along with health condition indicators (i.e. blood pressure, heart rate, weight, BMI, body fat, waist, arm, and thigh circumference) were measured and tested at baseline and 3 months after the initiation of study treatment. All laboratory evaluations were performed by the Department of Laboratory Medicine of CSMUH.

### Compliance rate

Compliance rate was calculated using the formula: ‘the number of capsules taken by the subjects / the number of capsules should be taken by the subjects × 100%’.

### Subjective evaluations

Subjective self-evaluation of negative effect after the administration of the study product was obtained at every doctor visit or through telephone interview to monitor the safety of the investigational product. Each patient was carefully monitored for the development of adverse events by the physician.

### Statistical analysis

All data of individuals who have completed the study were entered for efficacy and safety analysis. Data were presented as mean and standard deviation for continuous variables, and frequency and percentage were presented for categorical variables. The Mann–Whitney U test was performed to compare the change from baseline for study assessments between the two groups, and the Wilcoxon signed-rank test was used to compare the values before and after ingestion for each group. Categorical variables were compared between the two groups using Fisher’s exact test, and the changes before and after ingestion within each group were analyzed using the McNemar test. All statistical analyses were performed using SPSS version 18.0 for Windows (SPSS Inc., Chicago, IL, USA), and the level of significance was set at 0.05.

## Results

### Demographic characteristics and concomitant medication

**Fig. 1:**
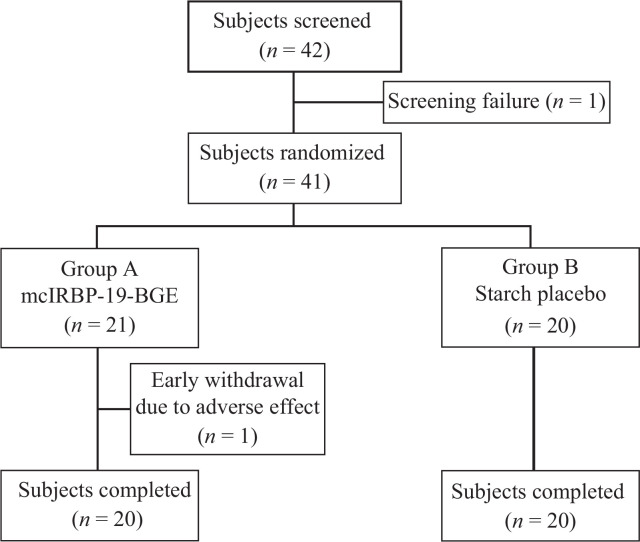
Disposition of the trial subjects.

Among 42 subjects who have been enrolled into the study, one subject was excluded before randomization due to the treatment goal being reached during the screening period, and one subject from mcIRBP-19-BGE group dropped out because of the occurrence of papule all over the body (Fig. 1). Of the 40 subjects who completed the study, 29 patients whose HbA1c did not continuously decline at the enrollment were used for subgroup analysis. Demographic characteristics and baseline hypoglycemic medications were compared between the two groups for all subjects and the subset individuals (Table 1). None of these variables were significantly different between the two treatment groups.

**Table 1:** Demographic characteristics and hypoglycemic medication of study subjects

Assessments	All subjects				Subset subjects			
	mcIRBP-19-BGE (*n* = 20)		Placebo (*n* = 20)		mcIRBP-19-BGE (*n* = 14)		Placebo (*n* = 15)	
	*N*	%	*N*	%	*N*	%	*N*	%
**Gender**								
Male	6	30.0	5	25.0	4	28.6	4	26.7
Females	14	70.0	15	75.0	10	71.4	11	73.3
Age (mean ± SD)	58.3 ± 12.7		58.6 ± 13.9		61.1 ± 9.7		56.8 ± 15.4	
**Hypoglycemic medication**								
SU/non-SU	14	70.0	15	75.0	8	57.1	11	78.6
Metformin	18	90.0	20	100.0	12	85.7	15	100.0
TZD	5	25.0	3	15.0	3	21.4	2	14.3
DPP-4 inhibitor	9	45.0	10	50.0	7	50.0	7	50.0
SGLT2 inhibitor	5	25.0	9	45.0	3	21.4	7	14.0
α-Glucosidase inhibitor	1	5.0	0	0.0	1	7.1	0	0.0

No significant differences between the two groups were observed for all variables. The subset subjects mean that those subjects whose HbA1c levels showed continued decline before the enrollment for 3 months were excluded.

The oral hypoglycemic drugs were classified as 1) insulin secretagogues (e.g. sulfonylurea [SU] and non-sulfonylurea [non-SU]), 2) insulin sensitizers (e.g. metformin, thiazolidinedione [TZD]), 3) α-glucosidase inhibitors, 4) dipeptidyl peptidase-4 (DPP-4) inhibitors, and 5) sodium glucose cotransporter 2 (SGLT2) inhibitors (24). Almost all the subjects (90% for the mcIRBP-19-BGE group and 100% for the placebo group) had taken metformin, about 70–75% of the subjects had taken SU and non-SU, followed by DPP-4 inhibitor, SGLT2 inhibitor, and TZD (Table 1).

### Baseline assessments and compliance rate

There were no significant differences in the baseline assessments, including glycemic indices, total protein, uric acid, liver and kidney function, vital signs, body weight, BMI, body fat, and circumferences of waist, hip, arm, and thigh between the two groups for both analysis sets (Table 2). The compliance rates were 94.3 and 93.2% for all subjects and the subset individuals, respectively.

**Table 2:** Baseline assessments

Assessments	All subjects					Subset subjects				
	mcIRBP-19-BGE (*n* = 20)		Placebo (*n* = 20)		*P*	mcIRBP-19-BGE (*n* = 14)		Placebo (*n* = 15)		*P*
	Mean	SD	Mean	SD		Mean	SD	Mean	SD	
**Glycemic assessments**										
Fasting glucose (mg/dL)	172.5	30.9	169.4	27.5	0.655	176.5	32.6	166.5	26.4	0.315
HbA1c (%)	7.8	0.7	7.9	0.6	0.616	8.0	0.7	8.0	0.7	0.930
**Safety assessments**										
AST (U/L)	26.7	10.9	24.2	8.6	0.385	29.4	11.9	26.0	9.1	0.335
ALT (U/L)	28.5	14.2	25.1	11.0	0.542	30.7	15.2	27.9	11.3	0.861
BUN (mg/dL)	14.2	3.9	17.7	8.0	0.210	14.6	3.6	18.1	8.8	0.381
creatinine (mg/dL)	0.8	0.2	0.9	0.3	0.409	0.8	0.2	0.9	0.3	0.662
Uric acid (mg/dL)	5.6	1.5	6.0	1.8	0.533	5.7	1.7	6.3	1.8	0.541
Hb (g/dL)	14.4	1.4	14.0	1.0	0.432	14.4	1.6	14.0	1.1	0.541
**Health conditions**										
Body weight (kg)	67.1	13.9	66.8	13.5	0.968	67.7	14.7	69.6	13.3	0.541
BMI (kg/m^2^)	26.0	4.2	26.3	4.5	0.850	26.0	4.5	27.4	4.4	0.359
Waist circumference (cm)	92.1	10.9	90.4	9.4	0.534	92.7	12.3	91.9	8.8	0.727
Hip circumference (cm)	100.0	9.0	99.3	8.8	0.935	100.1	9.6	100.9	8.3	0.727
Arm circumference (cm)	30.4	4.0	31.6	3.8	0.310	30.5	4.1	32.2	3.3	0.182
Thigh circumference (cm)	47.3	5.4	48.8	6.7	0.675	48.1	4.9	49.9	7.3	0.600
Body fat (%)	32.2	5.5	32.9	5.6	0.685	31.9	4.9	33.0	5.5	0.600
Total protein (mg/dL)	7.3	0.4	7.2	0.4	0.540	7.3	0.4	7.2	0.4	0.510
Systolic blood pressure (SBP) (mmHg)	136.6	17.9	136.7	17.2	0.903	136.3	20.0	138.9	15.1	0.710
Diastolic blood pressure (DBP) (mmHg)	78.6	8.2	77.1	10.5	0.542	78.1	9.8	79.3	10.7	0.743
Heart rate (bpm)	82.6	10.4	79.9	8.2	0.481	84.2	10.5	78.5	6.2	0.156

Body mass index = body weight (kg) / height (m)^2^.

The subset subjects mean that those subjects whose HbA1c levels showed continued decline before the enrollment for 3 months were excluded.

### Before and after treatment comparison

Tables 3 and 4 summarizes the comparison results for placebo and mcIRBP-19-BGE groups before and after treatment, respectively. For all subject analysis, FBG and HbA1c decreased with a borderline significance (*P* = 0.057 and 0.060 for FBG and HbA1c, respectively) for the mcIRBP-19-BGE group (Table 4); however, no significant changes (*P* > 0.05) were observed for the placebo group (Table 3). For the subset analysis, FBG and HbA1c significantly decreased (from 176.5 ± 32.6 to 159.4 ± 18.3, *P* = 0.041 for FBG and from 8.0 ± 0.7 to 7.5 ± 0.8, *P* = 0.010 for HbA1c) for the mcIRBP-19-BGE group (Table 4); however, no significant changes (*P* = 0.776 and 0.608 for FBG and HbA1c, respectively) were observed for the placebo group (Table 3). The results showed a significant reducing effect on FBG and HbA1c in the subset analysis but only with borderline significance in the analysis of all subjects.

**Table 3:** The comparison of assessments before and after study treatment for the placebo group

Assessments	All subjects in placebo group (*n* = 20)					Subset subjects in placebo group (*n* = 15)				
	Before		After		*P*	Before		After		*P*
	Mean	SD	Mean	SD		Mean	SD	Mean	SD	
Fasting glucose (mg/dL)	169.4	27.5	158.9	32.8	0.263	166.5	26.4	159.9	34.5	0.776
HbA1c (%)	7.9	0.6	8.0	1.0	0.779	8.0	0.7	8.1	1.1	0.608
HbA1c < 7% (*n*, %)	0, 0.0%		2, 10.0%		0.500	0, 0.0%		2, 13.3%		0.500
BMI (kg/m^2^)	26.3	4.5	26.1	4.5	0.122	27.4	4.4	27.1	4.6	0.140
Body weight (kg)										
Month 1	66.8	13.5	66.8	14.0	0.736	69.6	13.3	69.7	14.0	0.972
Month 3	66.8	13.5	66.3	13.9	0.163	69.6	13.3	69.1	14.1	0.198
Waist circumference (cm)	90.4	9.4	91.3	8.7	0.275	91.9	8.8	92.9	8.3	0.329
Hip circumference (cm)	99.3	8.8	98.9	8.9	0.493	100.9	8.3	100.6	8.9	0.562
Arm circumference (cm)	31.6	3.8	30.4	3.7	**0. 006**	32.2	3.3	31.3	3.3	**0.025**
Thigh circumference (cm)	48.8	6.7	47.9	6.4	**0.030**	49.9	7.3	49.0	6.8	0.063
Body fat (%)	32.9	5.6	32.7	6.3	0.654	33.0	5.5	32.8	6.5	0.820
Total protein (mg/dL)	7.2	0.4	7.4	0.5	**0.012**	7.2	0.4	7.4	0.4	0.063
SBP (mmHg)										
Month 1	136.7	17.2	132.9	15.6	0.067	138.9	15.1	133.5	13.7	0.068
Month 3	136.7	17.2	132.8	12.9	0.198	138.9	15.1	133.8	12.0	0.124
DBP (mmHg)										
Month 1	77.1	10.5	74.6	9.5	0.153	79.3	10.7	76.5	10.3	0.148
Month 3	77.1	10.5	75.2	11.3	0.190	79.3	10.7	78.3	11.2	0.550
Heart rate (bpm)										
Month 1	79.9	8.2	82.0	10.7	0.276	78.5	6.2	80.7	11.3	0.363
Month 3	79.9	8.2	80.3	9.3	0.519	78.5	6.2	78.5	8.3	0.826

*P*-value by Wilcoxon signed-rank test or McNemar chi-square test when appropriate. The subset subjects mean that those subjects whose HbA1c levels showed continued decline before the enrollment for 3 months were excluded. *P* values less than 0.05 significance level are shown in bold.

**Table 4:** The comparison of assessments before and after study treatment for mcIRBP-19-BGE group

Assessments	All subjects in mcIRBP-19-BGE group (*n* = 20)					Subset subjects in mcIRBP-19-BGE group (*n* = 14)				
	Before		After		*P*	Before		After		*P*
	Mean	SD	Mean	SD		Mean	SD	Mean	SD	
Fasting glucose (mg/dL)	172.5	30.9	160.0	22.8	0.057	176.5	32.6	159.4	18.3	**0. 041**
HbA1c (%)	7.8	0.6	7.6	0.8	0.060	8.0	0.7	7.5	0.8	**0.010**
HbA1c <7% (*n*, %)	0, 0.0%		5, 25.0%		0.063	0, 0.0%		4, 28.6%		0.125
BMI (kg/m^2^)	26.0	4.2	25.9	4.2	0.526	26.0	4.5	25.8	4.4	0.272
Body weight (kg)										
Month 1	67.1	13.9	66.8	13.7	0.219	67.7	14.7	66.9	14.3	**0.042**
Month 3	67.1	13.9	66.9	13.9	0.538	67.7	14.7	67.2	14.3	0.272
Waist circumference (cm)	92.1	10.9	92.8	11.5	0.367	92.7	12.3	93.1	13.1	0.623
Hip circumference (cm)	100.0	9.0	100.0	8.9	0.680	100.1	9.6	100.5	9.1	0.925
Arm circumference (cm)	30.4	4.0	29.8	3.7	**0.044**	30.5	4.1	30.0	4.0	0.145
Thigh circumference (cm)	47.3	5.4	47.2	5.6	1.000	48.1	4.9	47.6	5.4	0.166
Body fat (%)	32.2	5.5	32.4	5.5	0.533	31.9	4.9	32.1	4.8	0.345
Total protein (mg/dL)	7.3	0.4	7.4	0.4	0.073	7.3	0.4	7.4	0.4	0.114
SBP (mmHg)										
Month 1	136.6	17.9	134.1	15.8	0.490	136.3	20.0	134.6	17.5	0.754
Month 3	136.6	17.9	129.7	13.8	0.144	136.3	20.0	128.0	13.5	0.117
DBP (mmHg)										
Month 1	78.6	8.2	78.2	7.6	0.708	78.1	9.8	78.1	6.4	0.925
Month 3	78.6	8.2	76.3	10.4	0.243	78.1	9.8	75.1	5.7	0.345
Heart rate (bpm)										
Month 1	82.6	10.4	82.2	11.1	0.360	85.4	10.2	81.9	10.0	**0.044**
Month 3	82.6	10.4	81.7	11.4	0.444	85.4	10.2	79.6	10.9	**0.028**

*P*-value by Wilcoxon signed-rank test or McNemar chi-square test when appropriate. The subset subjects mean that those whose HbA1c levels showed continued decline before the enrollment for 3 months were excluded. *P* values less than 0.05 significance level are shown in bold.

A significant reduction in the circumference of both arm and thigh (from 31.6 ± 3.8 to 30.4 ± 3.7, *P* = 0.006 for arm and from 48.8 ± 6.7 to 47.9 ± 6.4, *P* = 0.030 for thigh) was observed at month 3 for the placebo group (Table 3); but only the arm circumference was significantly decreased (from 30.4 ± 4.0 to 29.8 ± 3.7, *P* = 0.044) for the mcIRBP-19-BGE group (Table 4). As for the subset individuals, the placebo group showed a significant decrease (from 32.2 ± 3.3 to 31.3 ± 3.3, *P* = 0.025) in arm circumference (Table 3) but no significant changes (from 30.5 ± 4.1 to 30.0 ± 4.0, *P* = 0.145) in arm circumferences for the mcIRBP-19-BGE group (Table 4), which indicated that mcIRBP-19-BGE might delay the reduction of the arm or thigh circumference.

A significant reduction in heart rate was observed among the subset individuals after taking the mcIRBP-19-BGE capsules for 1 month (from 85.4 ± 10.2 to 81.9 ± 10.0, *P* = 0.044) and continued through 3 months (79.6 ± 10.9, *P* = 0.028) (Table 4), while no significant changes were observed for the placebo group (Table 3). However, such reduction in heart rate was not observed for either the placebo or mcIRBP-19-BGE group in the analysis of all subjects (Tables 3 and 4). The placebo group showed a significant increase in total protein at the end of the study (*P* = 0.012); however, the change was with a borderline significance for subset individuals (*P* = 0.063; Table 3). The mcIRBP-19-BGE subset group showed a significant decrease in body weight at month 1; however, the significant change no longer existed at month 3 (Table 4).

Before and after treatment comparison of the percentage of subjects reaching the HbA1c treatment goal was also performed. The results showed that the percentage of the subjects who reached the treatment goal (i.e. HbA1c < 7) increased from 0.0 to 10.0% (*P* = 0.500) after 3 months of administration for the placebo group in all subjects and from 0.0 to 13.3% (*P* = 0.500) in the subset, respectively (Table 3). For the mcIRBP-19-BGE group, the results revealed from 0.0 to 25.0% (*P* = 0.063) in all subjects and from 0.0 to 28.6% (*P* = 0.125) in the subset, respectively (Table 4). These results have suggested that the percentage for the subjects who reached the treatment goal was not significantly increased by the treatment of mcIRBP-19-BGE.

### The change from baseline evaluations

The change from baseline for most of the evaluations was not significantly different between the two groups (Supplementary Table 1 in Supplementary Material), except that after 3 months of administration there was a borderline significant difference at HbA1c between the two groups for subset individuals (−0.4 ± 0.5% for mcIRBP-19-BGE vs. 0.2 ± 0.8% for placebo, *P* = 0.051).

### Safety and subjective evaluations

All study subjects showed no significant changes in safety assessments. One of the subjects from the mcIRBP-19-BGE group had papule all over the body and dropped out of the trial. The condition did not subside after the cessation of the investigational product treatment, and the physician confirmed that the event was not caused by the treatment.

## Discussion

The purpose of this study was to investigate whether the mcIRBP-19-BGE had a hypoglycemic effect in type 2 diabetic patients who had taken antidiabetic medication but failed to achieve the treatment goal. This study was also intended to reveal the hypoglycemic efficacy of mcIRBP-19-BGE when the antidiabetic medications were ineffective. Not fully in line with our hypothesis, we found from the analysis results of all subjects that mcIRBP-19-BGE for patients with the antidiabetic medications only had a marginal hypoglycemic efficacy. However, the results from the subset analysis showed that oral administration of mcIRBP-19-BGE could significantly reduce FBG and HbA1c for those who failed to respond to the antidiabetic medications. All of these results have demonstrated that mcIRBP-19-BGE had a hypoglycemic effect, and could have a significant reduction in FBG and HbA1c for the type 2 diabetic patients when the hypoglycemic medications were ineffective. To the best of our knowledge, this study is the first reported on studying the hypoglycemic efficacy of mcIRBP-19-BGE in human subjects.

It had been reported that taking SU for 3–6 months could cause 1.0–1.5% decrease in HbA1c , 1.0–1.5% in repaglinide (a non-SU), 0.5–1.0% in nateglinide (a non-SU), 1.0–1.5% in metformin, 0.5–1.5% in TZD, 0.5–1.5% in α-glucosidase inhibitor, 0.4–1.1% in DPP-4 inhibitor, and 0.39–2.05% in SGLT2 inhibitor (24–26). The present results showed that oral administration of mcIRBP-19-BGE capsules (600 mg/day) for 3 months could decrease HbA1c approximately 0.5% in the subset individuals whose hypoglycemic medications showed no effects to continuously decrease HbA1c. At such a condition, subjects of mcIRBP-19-BGE group (combined administration of hypoglycemic medications and mcIRBP-19-BGE) demonstrated a significant reduction in both FBG and HbA1c. The result has illustrated that the hypoglycemic efficacy was mainly from mcIRBP-19-BGE for the patients who failed to respond to the antidiabetic medication. However, mcIRBP-19 apparently is only a minor ingredient and other ingredients in the water extract of *Momordica charantia* might have also contributed to the beneficial effects observed, as reported in many literatures.

We wondered whether mcIRBP-19-BGE had a significant hypoglycemic effect on the type 2 diabetic patients who had failed to have the drug efficacy by the antidiabetic medications. According to the results from this study, mcIRBP-19-BGE at the used dosage (i.e. 600 mg/kg) could reduce blood glucose only for the subset patients who could not benefit from the hypoglycemic drugs. However, mcIRBP-19-BGE at the used dosage did not significantly decrease both HbA1c and FBG from the analysis of all subjects, suggesting that the hypoglycemic efficacy of mcIRBP-19-BGE was low for all of the type 2 diabetic patients who had taken antidiabetic medication but failed to achieve the treatment goal. However, the marginal hypoglycemic efficacy of mcIRBP-19-BGE for all subjects might be attributed to the interference from antidiabetic drugs. Because some subjects showed a steady decrease in HbA1c before the initiation of the study treatment (i.e. the drugs were still effective for these subjects), the hypoglycemic efficacy of mcIRBP-19-BGE may be interfered by the drug efficacy for both treatment and placebo groups at this situation. Another possible explanation for the marginal hypoglycemic efficacy of mcIRBP-19-BGE for all subjects might be due to the dose used. In this study, a dose of about 2.82 times that used in the previous animal study (18) may be insufficient for the human subjects. Another clinical trial is warranted to reveal the hypoglycemic efficacy of mcIRBP-19-BGE at a higher dose for the type 2 diabetic patients with failure in the drug treatment.

Moreover, this study found that the diabetic patients had a decrease in the circumference of arms and thighs, which might be related to the increasing risk of sarcopenia from the diabetes (27). Although the literature is not sufficient, insulin resistance may affect protein metabolism via affecting carbohydrate metabolism, thereby reducing protein synthesis, which may result in a loss of lean body tissue, especially muscle mass and muscle strength, thus leading to sarcopenia (28). At present, muscle mass can be estimated by measuring the arm muscle area, which is calculated by the mid-upper arm circumference and the triceps skinfold thickness (TSF) (29). Besides, the thigh circumference can reflect the muscle mass in the body (30). The results of this study revealed that taking mcIRBP-19-BGE could delay the reduction of circumferences of both arms and thighs in the subjects, suggesting that oral administration of mcIRBP-19-BGE may also benefit type 2 diabetic patients in terms of sarcopenia. In addition, the results clearly showed that mcIRBP-19-BGE had the effect of regulating heart rate in the subset patients. To the best of our knowledge, there is no study on the functions of *Momordica charantia* relating to regulating heart rate in the literature. The regulating heart rate in subset patients but not in all study subjects implied that the efficacy of mcIRBP-19-BGE to regulate heart rate might be related to the hypoglycemic medications. The mechanism associated with the slowing in the heart rate by mcIRBP-19-BGE still needs further investigations. Moreover, the mechanism for the significant body weight decrease and the total protein increase after taking mcIRBP-19-BGE for 1 month for the subgroup analysis were not clear.

In summary, this study has shown that mcIRBP-19-BGE can significantly reduce FBG and HbA1c levels for the patients who failed to respond to the antidiabetic medications. The hypoglycemic efficacy of mcIRBP-19-BGE approximately decreased HbA1c by 0.5%, on average, for a 3-month administration at a dose of 600 mg/day for the subset patients. All of these results have suggested that mcIRBP-19-BGE can be an alternative treatment option for the type 2 diabetic patients when the antidiabetic drugs are ineffective. The mcIRBP-19-BGE may also have the effect of preventing the decrease of the arm and thigh circumferences, and regulating the heart rate of the type 2 diabetic patients. Another clinical trial with a higher dose of mcIRBP-19-BGE is highly recommended for the further investigations.

## References

[1] International Diabetes Federation. IDF Diabetes Atlas. 8th ed. 2017. Available from: http://www.diabetesatlas.org/ [cited 28 April 2017].

[2] American Diabetes Association. 6. Glycemic targets: standards of medical care in diabetes – 2020. Diabetes Care 2020; 43(Suppl 1): S66–76. doi: 10.2337/dc20-S006 31862749

[3] Wallia A, Molitch ME. Insulin therapy for type 2 diabetes mellitus. JAMA 2014; 311: 2315–25. doi: 10.1001/jama.2014.5951 24915263

[4] Avilés-Santa L, Sinding J, Raskin P. Effects of metformin in patients with poorly controlled, insulin-treated type 2 diabetes mellitus. A randomized, double-blind, placebo-controlled trial. Ann Intern Med 1999; 131: 182–8. doi: 10.7326/0003-4819-131-3-199908030-00004 10428734

[5] Nakar S, Yitzhaki G, Rosenberg R, Vinker S. Transition to insulin in Type 2 diabetes: family physicians’ misconception of patients’ fears contributes to existing barriers. J Diabetes Complications 2007; 21: 220–6. doi: 10.1016/j.jdiacomp.2006.02.004 17616351

[6] Polonsky WH, Fisher L, Guzman S, Villa-Caballero L, Edelman SV. Psychological insulin resistance in patients with type 2 diabetes: the scope of the problem. Diabet Care 2005; 28: 2543–45. doi: 10.2337/diacare.28.10.2543 16186296

[7] Leung L, Birtwhistle R, Kotecha J, Hannah S, Cuthbertson S. Anti-diabetic and hypoglycaemic effects of Momordica charantia (bitter melon): a mini review. Br J Nutr 2009; 102: 1703–8. doi: 10.1017/S0007114509992054 19825210

[8] Fuangchan A, Sonthisombat P, Seubnukarn T, Chanouan R, Chotchaisuwat P, Sirigulsatien V, et al. Hypoglycemic effect of bitter melon compared with metformin in newly diagnosed type 2 diabetes patients. J Ethnopharmacol 2011; 134: 422–28. doi: 10.1016/j.jep.2010.12.045 21211558

[9] Singh J, Cumming E, Manoharan G, Kalasz H, Adeghate E. Medicinal chemistry of the anti-diabetic effects of Momordica Charantia: active constituents and modes of actions. Open Med Chem J 2011; 5(suppl2): 70–77. doi: 10.2174/1874104501105010070 21966327 PMC3174519

[10] Joseph B, Jini D. Antidiabetic effects of Momordica charantia (bitter melon) and its medicinal potency. Asian Pac J Trop Dis 2013; 3: 93–102. doi: 10.1016/S2222-1808(13)60052-3

[11] Tan MJ, Ye JM, Turner N, Hohnen-Behrens C, Ke CQ, Tang CP, et al. Antidiabetic activities of triterpenoids isolated from bitter melon associated with activation of the AMPK pathway. Chem Biol 2008; 15: 263–73. doi: 10.1016/j.chembiol.2008.01.013 18355726

[12] Iseli TJ, Turner N, Zeng XY, Cooney GJ, Kraegen EW, Yao S, et al. Activation of AMPK by bitter melon triterpenoids involves CaMKKβ. PLoS One 2013; 8: e62309. doi: 10.1371/journal.pone.0062309 23638033 PMC3636144

[13] Khanna P, Jain SC, Panagariya A, Dixit VP. Hypoglycemic activity of polypeptide-p from a plant source. J Nat Prod 1981; 44: 648–55. doi: 10.1021/np50018a002 7334382

[14] Liu SX, Fu ZP, Mu RM, Hu ZB, Wang FJ, Wang XR. Expression and characterization of Momordica Chanrantia anti-hyperglycaemic peptide in Escherichia coli. Mol Biol Rep 2010; 37: 1781–6. doi: 10.1007/s11033-009-9609-0 19585270

[15] Rajasekhar MD, Badri KR, Vinay Kumar K, Babu KR, Fatima SS, Sampath Kumar MT, et al. Isolation and characterization of a novel antihyperglycemic protein from the fruits of Momordica cymbalaria. J Ethnopharmacol 2010; 128: 58–62. doi: 10.1016/j.jep.2009.12.025 20038451

[16] Lo HY, Ho TY, Lin C, Li CC, Hsiang CY. Momordica charantia and its novel polypeptide regulate glucose homeostasis in mice via binding to insulin receptor. J Agric Food Chem 2013; 61: 2461–8. doi: 10.1021/jf3042402 23414136

[17] Lo HY, Ho TY, Li CC, Chen JC, Liu JJ, Hsiang CY. A novel insulin receptor-binding protein from Momordica charantia enhances glucose uptake and glucose clearance in vitro and in vivo through triggering insulin receptor signaling pathway. J Agric Food Chem 2014; 62: 8952–61. doi: 10.1021/jf5002099 25144709

[18] Lo HY, Li CC, Ho TY, Hsiang CY. Identification of the bioactive and consensus peptide motif from Momordica charantia insulin receptor-binding protein. Food Chem 2016; 204: 298–305. doi: 10.1016/j.foodchem.2016.02.135 26988505

[19] Lo HY, Li CC, Chen FY, Chen JC, Hsiang CY, Ho TY. Gastro-resistant insulin receptor-binding peptide from Momordica charantia improved the glucose tolerance in streptozotocin-induced diabetic mice via insulin receptor signaling pathway. J Agric Food Chem 2017; 65: 9266–74. doi: 10.1021/acs.jafc.7b03583 28994284

[20] Efird JT, Choi YM, Davies SW, Mehra S, Anderson EJ, Katunga LA. Potential for improved glycemic control with dietary Momordica charantia in patients with insulin resistance and pre-diabetes. Int J Environ Res Public Health 2014; 11: 2328–45. doi: 10.3390/ijerph110202328 24566057 PMC3945602

[21] Dans AM, Villarruz MV, Jimeno CA, Javelosa MA, Chua J, Bautista R, et al. The effect of Momordica charantia capsule preparation on glycemic control in type 2 diabetes mellitus needs further studies. J. Clin Epidemiol 2007; 60: 554–9. doi: 10.1016/j.jclinepi.2006.07.009 17493509

[22] John AJ, Cherian R, Subhash HS, Cherian AM. Evaluation of the efficacy of bitter gourd (momordica charantia) as an oral hypoglycemic agent – a randomized controlled clinical trial. Indian J Physiol Pharmacol 2003; 47: 363–5. 14723327

[23] Zänker KS, Mang B, Wolters M, Hahn A. Personalized diabetes and cancer medicine: a rationale for anti-diabetic nutrition (Bitter Melon) in a supportive setting. Curr Cancer Ther Rev 2012; 8, 66–77. doi: 10.2174/157339412799462521

[24] Cheng AY, Fantus IG. Oral antihyperglycemic therapy for type 2 diabetes mellitus. CMAJ 2005; 172: 213–26. doi: 10.1503/cmaj.1031414 15655244 PMC543986

[25] Davis TM. Dipeptidyl peptidase-4 inhibitors: pharmacokinetics, efficacy, tolerability and safety in renal impairment. Diabetes Obes Metab 2014; 16: 891–9. doi: 10.1111/dom.12295 24684351

[26] Scheen AJ. Pharmacodynamics, efficacy and safety of sodium-glucose co-transporter type 2 (SGLT2) inhibitors for the treatment of type 2 diabetes mellitus. Drugs 2015; 75: 33–59. doi: 10.1016/10.1007/s40265-014-0337-y 25488697

[27] Shishikura K, Tanimoto K, Sakai S, Tanimoto Y, Terasaki J, Hanafusa T. Association between skeletal muscle mass and insulin secretion in patients with type 2 diabetes mellitus. Endocr J 2014; 61: 281–7. doi: 10.1507/endocrj.EJ13-0375 24420336

[28] Morais JA, Jacob KW, Chevalier S. Effects of aging and insulin resistant states on protein anabolic responses in older adults. Exp Gerontol 2018; 108: 262–8. doi: 10.1016/j.exger.2018.04.025 29723655

[29] Heymsfield SB, McManus C, Smith J, Stevens V, Nixon DW. Anthropometric measurement of muscle mass: revised equations for calculating bone-free arm muscle area. Am J Clin Nutr 1982; 36: 680–90. doi: 10.1093/ajcn/36.4.680 7124671

[30] Kwon HR, Han KA, Ahn HJ, Lee JH, Park GS, Min KW. The correlations between extremity circumferences with total and regional amounts of skeletal muscle and muscle strength in obese women with type 2 diabetes. Diabetes Metab J 2011; 35: 374–83. doi: 10.4093/dmj.2011.35.4.374 21977457 PMC3178698

